# EXPLORING THE WITHIN-PERSON COUPLING RELATIONSHIP BETWEEN BLOOD PRESSURE AND COGNITION IN OLDER AFRICAN AMERICANS

**DOI:** 10.1093/geroni/igz038.2412

**Published:** 2019-11-08

**Authors:** DeAnnah R Byrd, Alyssa A Gamaldo, Jason C Allaire, Keith E Whitfield

**Affiliations:** 1 Wayne State University, Detroit, Michigan, United States; 2 Penn State, University Park, Pennsylvania, United States; 3 NC State University, Raleigh, North Carolina, United States

## Abstract

We examined the within-person relationship between systolic blood pressure (SBP) and cognition and whether this association is moderated by hypertension status and stress level. Analysis was conducted on 50 (39 women and 11 men) community-dwelling African Americans ranging in age from 50 to 80 years (M = 65.40, SD = 8.53). Participants’ blood pressure and cognition (i.e., executive function, memory, perceptual speed, inductive reasoning, constructional praxis, and language) was assessed on 8 different occasions over a 2-week time period. Stress was measured at baseline using the Elderly Life Stress Inventory. Findings show on days systolic blood pressure increased above an individual’s average level, their memory performance also tended to improve on that day. A significant 3-way interaction was further observed; such that on occasions when an individual’s systolic blood pressure was above his or her personal average, their executive functioning performance (i.e., Letter Fluency test) was significantly better, particularly for participants who on average had a high SBP (or were hypertensive) and reported a history of high stress. These results suggest that higher levels of blood pressure are related to better cognitive performance among middle age to older African Americans. These counterintuitive findings may be attributed to the inclusion of a selective sample of resilient African Americans who have survived despite having adverse health conditions. This association may also arise from stress induced high effort coping, which is needed to perform well on cognitive tasks but also increases blood pressure.

